# Beyond Lipoprotein(a) plasma measurements: Lipoprotein(a) and inflammation

**DOI:** 10.1016/j.phrs.2021.105689

**Published:** 2021-05-23

**Authors:** Gissette Reyes-Soffer, Marit Westerterp

**Affiliations:** a Columbia University Medical Center College of Physicians and Surgeons, Department of Medicine, Division of Preventive Medicine and Nutrition, New York, NY, USA; b Department of Pediatrics, University Medical Center Groningen, University of Groningen, Groningen, The Netherlands

**Keywords:** Inflammation, Atherosclerosis, Lipoprotein(a), Cardiovascular diseases

## Abstract

Genome wide association, epidemiological, and clinical studies have established high lipoprotein(a) [Lp(a)] as a causal risk factor for atherosclerotic cardiovascular disease (ASCVD). Lp(a) is an apoB100 containing lipoprotein covalently bound to apolipoprotein(a) [apo(a)], a glycoprotein. Plasma Lp(a) levels are to a large extent determined by genetics. Its link to cardiovascular disease (CVD) may be driven by its pro-inflammatory effects, of which its association with oxidized phospholipids (oxPL) bound to Lp(a) is the most studied. Various inflammatory conditions, such as rheumatoid arthritis (RA), systemic lupus erythematosus, acquired immunodeficiency syndrome, and chronic renal failure are associated with high Lp(a) levels. In cases of RA, high Lp(a) levels are reversed by interleukin-6 receptor (IL-6R) blockade by tocilizumab, suggesting a potential role for IL-6 in regulating Lp(a) plasma levels. Elevated levels of IL-6 and IL-6R polymorphisms are associated with CVD. Therapies aimed at lowering apo(a) and thereby reducing plasma Lp(a) levels are in clinical trials. Their results will determine if reductions in apo(a) and Lp(a) decrease cardiovascular outcomes. As we enter this new arena of available treatments, there is a need to improve our understanding of mechanisms. This review will focus on the role of Lp(a) in inflammation and CVD.

## 1. Introduction

Genome wide association, epidemiological, and clinical studies have established high lipoprotein(a) [Lp(a)] as a causal risk factor for atherosclerotic cardiovascular disease (ASCVD) [1,2]. Lp(a) contains several proteins [3]. Its main protein components are apolipoprotein(a) [apo(a)] and apolipoprotein B100 (apoB100) which are bound by covalent and non-covalent bonds [4]. Lp(a) also contains cholesterol, phospholipids, and triglycerides [5]. High plasma levels of Lp(a) are linked to atherosclerosis, thrombosis, and arterial calcification [6], which may be driven by its pro-inflammatory effects [7]. Lp(a) has been found in atherosclerotic plaques [8]. Several studies have suggested that Lp(a) itself may enhance inflammation in endothelial cells, monocytes, and macrophages, via the oxidized phospholipids (oxPL) that are bound to Lp(a) [9–11]. Previous studies point to both the apoB component and the apo(a) moiety as drivers of inflammation that can result in ASCVD [3,12]. Proteomic studies have also identified a large number of apolipoproteins on Lp(a), as well as platelet-activating acetylhydrolase (PAF-AH) and paraoxinase-1 (PON-1) [3,13,14], which may link Lp(a) to disease states. Some currently identified proteins and their function are listed in Table 1.

Plasma Lp(a) levels are to a large extent determined by genetics [15,16]. The *LPA* gene is derived from a duplication of the plasminogen gene, but apo(a) has no plasminogen-like activity and so, in addition to its apoB containing lipoprotein structure, it may compete with plasminogen for binding to fibrin, thereby impairing fibrinolysis and promoting thrombosis at sites of endothelial breakdown [17,18]. Recent data suggest *ex vivo* measures of fibrinolysis are not affected in subjects with high Lp(a). However, *in vivo* studies are lacking [19].

High Lp(a) levels have also been reported in various inflammatory conditions, such as rheumatoid arthritis (RA), systemic lupus erythematosus, acquired immunodeficiency syndrome, chronic renal failure, and pulmonary arterial hypertension [20–31]. Differences in Lp(a) levels have also been observed in pregnancy [32,33], and diabetes [34,35], inflammatory conditions that will not be addressed in this review.

Large data sets pointing to Lp(a) as a top genetic marker for cardiovascular disease [36,37] have increased interest in the identification of pathways and involved culprits of disease. Importantly, there are various treatments that lower Lp(a) levels. Non-lipid altering treatments affect Lp(a) levels, including niacin [1,6,11,38–40]. PCSK9 inhibitors modestly lower Lp(a) levels [9,41], as do additional apoB lowering therapies [42,43]. Apharesis lowers Lp(a) and other apoB containing lipoproteins, and is FDA approved for severe elevations of Lp(a) levels [11,44]. Studies assessing risk reduction of apharesis are ongoing (NCT02791802). There are three ongoing studies examining the effect of apo(a) lowering with novel targeted therapies (NCT04606602-SLN360, NCT04023552-TQJ230, and NCT04270760-AMG 890) [10,45–49]. As we enter a new arena of available treatments, there is a need to improve our understanding of mechanisms downstream of Lp(a) and mechanisms that regulate plasma Lp(a) levels. This review will focus on the role of Lp(a) in inflammation and cardiovascular disease (CVD).

## 2. Inflammation and CVD

The CANTOS (canakinumab anti-inflammatory thrombosis outcomes study) trial has for the first time provided direct evidence that inflammation enhances CVD events in humans, by showing that antagonism of interleukin (IL)-1β decreases the incidence of recurrent CVD events in patients with high levels of C reactive protein (CRP) [50], irrespective of effects on plasma lipid levels. IL-1β is a main regulator of inflammation and cytokine secretion, including IL-6 [51,52]. Mendelian Randomization studies and meta-analyses have shown a link between IL-6R polymorphisms and CVD [53,54]. In a subgroup of CANTOS participants screened for IL-6 plasma levels (n = 4833), decreases in plasma levels of IL-6 downstream of IL-1β antagonism by canakinumab were associated with a reduction of CVD events [55]. Moreover, the residual CVD risk in this subgroup of the CANTOS trial was proportional to plasma IL-6 levels [56]. Together, these studies indicate an important role for IL-6 in CVD events, independent of plasma lipid levels. Other trials evaluating anti-inflammatory drugs (methotrexate and colchicine) in CVD include the CIRT (cardiovascular inflammation reduction trial), LoDoCo (low dose colchicine), COLCOT (colchicine cardiovascular outcomes trial), and LoDoCo2 (low dose colchicine 2) trials. While low-dose methotrexate was ineffective in reducing CVD events, leading to CIRT being stopped prematurely, the effects of colchicine on CVD are still being evaluated in ongoing research studies [57–61]. The outcome of these trials has recently been reviewed [62].

The above observations suggest that IL-6R blockade by tocilizumab, which is used as a therapy for RA, would have beneficial effects in CVD. However, tocilizumab treatment increases plasma LDL-cholesterol in RA patients [20,63–67], which has been attributed to decreased hepatic LDL receptor levels, as shown in HepG2 cells [65]. In three USA databases, as well as in studies in Japan and Italy, treatment with tocilizumab had no effects on major adverse cardiovascular events (MACE) [68–71]. Perhaps this was due to the study populations mainly consisting of RA patients that generally have higher levels of inflammation and lower LDL-cholesterol levels [63,72]. Two small trials assessed the role of tocilizumab in myocardial infarction (MI) directly, in patients that did not have RA. A clinical trial in non-ST-elevation myocardial infarction (nSTEMI) patients showed that tocilizumab did not affect MI [73] and the ASSAIL-MI (ASSessing the Effect of Anti-IL-6 Treatment in Myocardial Infarction) trial (NCT03004703) testing a role for tocilizumab in STEMI patients is ongoing [74]. In sum, the CANTOS trial has provided direct evidence for a role of inflammation in CVD, which has mainly been attributed to decreases in plasma IL-6 downstream of IL-1β signaling. A role for IL-6 in CVD is supported by Mendelian Randomization studies and meta-analyses showing links between IL-6R polymorphisms and CVD. Whether IL-6R blockade by tocilizumab decreases the incidence of CVD has only been studied in one small population of MI patients. The ASSAIL-MI trial, which is currently ongoing, is likely to provide additional insights as to whether tocilizumab decreases the incidence of CVD.

## 3. IL-6 regulates Lp(a) plasma levels

As previously stated, in addition to genetic control [15,16], various inflammatory diseases including RA have been linked to high Lp(a) levels. Studies using drugs to target inflammatory pathways in RA patients have provided insights into these links. IL-6R blockade by tocilizumab, but not tumor necrosis factor α (TNFα) blockade by adalimumab, decrease plasma Lp(a) levels by ~30–40% in RA patients [20,63,64,75]. These data thus suggest that decreased IL-6R signaling and not TNFα signaling, reduces plasma Lp(a) levels. Conversely, the IL-6 promoter polymorphism (−174 G/C), which is associated with high plasma IL-6 levels, correlates positively with plasma Lp(a) [76], as do plasma levels of IL-6 as demonstrated in ~1153 human subjects [77]. The latter was confirmed in a subgroup of subjects (n = 635) without chronic inflammatory disease, suggesting a positive relationship between plasma IL-6 and Lp(a) in the general population [77]. This subgroup showed a positive correlation of IL-6 responsive genes with *LPA* mRNA expression in liver biopsies, further supporting a positive association between IL-6 and Lp(a) [77]. *In vitro* experiments in hepatocytes transfected with a plasmid for *LPA* substantiated that IL-6 enhances *LPA* expression, mediated by binding of STAT3 to the *LPA* promoter [77]. Hence, multiple lines of evidence show regulation of plasma Lp(a) levels by IL-6.

However, a clinical trial in nSTEMI patients showed that tocilizumab did not affect Lp(a) levels, measured at day 1, 3, and months 3 and 6 after tocilizumab treatment [73]. Statin-therapy, which may increase Lp (a) levels, was suggested to explain why tocilizumab did not decrease Lp (a) levels in this specific cohort [73]. Indeed, a recent meta-analysis from six randomized trials (n = 5256 patients) using a single well-established method for Lp(a) measurements has shown that statins increase Lp(a) levels (11.6–20.4% for pravastatin and 18.7–24.2% for atorvastatin) [78]. Mechanistically, atorvastatin increased *LPA* mRNA levels and apolipoprotein(a) in HepG2 cells [78], via an as yet unidentified mechanism. In summary, signaling downstream of the IL-6R affects Lp(a) levels in RA patients. Whether this is also the case for CVD remains to be elucidated, although one study found no differences, perhaps because the tocilizumab effect on Lp(a) is compromised by statin-therapy. Nonetheless, the statin effects may not be the complete explanation for this outcome. In future studies, it would be of interest to evaluate whether IL-6R polymorphisms that associate with CVD risk affect plasma Lp(a) levels.

Apart from inflammation, Lp(a) inversely correlates with levels of bile acids in plasma in patients with biliary obstructions [79]. Mechanistic studies in animal models and hepatocytes revealed that activation of the Farnesoid X Receptor (FXR) by bile acids suppressed *LPA* mRNA transcription, irrespective of effects on inflammatory gene expression[79]. In support of these findings, a later study showed that therapy with the FXR agonist chenodeoxycholic acid (CDCA) for gallstone disease over a period of three weeks decreased plasma Lp(a) levels significantly [80]. In sum, both IL-6 and FXR pathways have been shown to regulate plasma Lp(a) levels.

## 4. Tocilizumab, Lp(a), and COVID-19 (SARS-CoV 2)

IL-6 is elevated during the cytokine storm in COVID-19 patients [81–83]. It has been hypothesized that Lp(a) levels, as a result thereof, may also be upregulated, and that high Lp(a) could contribute to inflammation and thrombosis observed in COVID-19 [84]. Importantly, studies in 9005 UK Biobank participants found that plasma levels of apoB, a component of Lp(a), was not associated with COVID-19 [85]. The link between IL-6, Lp(a), and COVID-19 is of interest, particularly in view of studies addressing whether tocilizumab suppresses symptoms and complications associated with COVID-19. Potential anti-inflammatory effects of tocilizumab could be mediated by decreases in Lp(a). The effect of tocilizumab on reducing symptoms and mortality related to COVID-19 has been investigated extensively. Some studies, though small in terms of number of patients, have shown clinical benefit [81–83], while others did not [86–88]. A recent meta-analysis and meta-regression has suggested that tocilizumab is associated with clinical meaningful improvements in COVID-19 [89]. This is supported by recent results in a larger group of 4116 COVID19 patients in the RECOVERY (Randomised Evaluation of COVID-19 Therapy) trial, that, although preliminary, showed that tocilizumab improves survival in hospitalized COVID19 patients with hypoxia and systemic inflammation [90]. Lp(a) may be elevated during the cytokine storm in COVID-19 and could contribute to an increased incidence of thrombosis [84].

## 5. Lp(a), oxidized phospholipids, and CVD risk

Lp(a) has been found to enhance inflammation, presumably due to oxidized phospholipids (oxPL) bound to Lp(a). In addition to Lp(a), oxPL circulate on oxidized LDL (oxLDL) and apoptotic cells [91]. This was found using E06, an antibody that binds to the phosphocholine head-group of most oxPL, but not PL [91]. E06 blocks the uptake of oxLDL, but not LDL by macrophages, as well as their ingestion of apoptotic cells, but not viable cells [91]. A landmark study by the Witztum Laboratory has shown that E06 suppresses inflammation and atherosclerosis in *Ldlr*^−/−^ mice, providing direct evidence that oxPL, in mice either derived from oxLDL or apoptotic cells, accelerates atherosclerosis [92].

Using the E06 and apoB-100/E06 assays, several studies have shown a positive relationship between oxPL and acute coronary syndrome (ACS), CVD events, and MI [93–95], indicating that oxPL is a clear pro-atherogenic factor in humans. Whether the positive relationship between oxPL and CVD is driven by oxPL bound to LDL and/or Lp(a), has been reviewed in detail [93]. A positive correlation between Lp(a) and plasma oxPL in plasma of patients from several CVD cohorts support a role of oxPL association with Lp(a) [93].

Lp(a) concentrations correlate inversely with the size of the apo(a) isoform, and the small apo(a) isoform has high affinity for oxPL [93,96,97]. This may explain the higher affinity of apo(a) for oxPL when Lp(a) concentrations are high [7,96,98,99]. Mendelian Randomization studies have shown that the small isoform of apo(a) associated with Lp(a) plasma levels > 50 mg/dL, increases CVD risk by 2–2.5-fold compared to the large apo(a) isoform [36,100]. These studies are limited to white cohorts and Lp(a) levels vary by ethnicity [101,102]. Importantly, not all subjects with Lp(a) plasma levels > 50 mg/dL develop CVD, suggesting that additional factors may be required for CVD to develop in the setting of high plasma Lp(a) levels.

Inflammation has been suggested to potentiate CVD risk mediated by Lp(a) and oxPL [103]. This was investigated by assessing the correlation between Lp(a) or apoB-bound-oxPL and CVD risk in carriers and non-carriers of an IL-1 haplotype that is associated with increased inflammation [103]. In this study a positive relationship between Lp(a) or apoB-bound-oxPL and CVD risk was shown in subjects carrying this IL-1 haplotype, but not in non-carriers [103]. This positive relationship was strengthened in subjects with high levels of CRP. These findings suggest that Lp(a) and oxPL enhance CVD in the presence of IL-1 induced inflammation, particularly when this results in high CRP levels.

## 6. Clinical implications of high Lp(a) linked to inflammation

The role of Lp(a) in inflammation has been evaluated extensively in *in vitro* studies. Early studies have shown that Lp(a) is a chemoattractant for monocytes and upregulates IL-6 secretion in these cells [104–106]. Lp(a) also enhances the expression of vascular cell adhesion molecule (VCAM)– 1, intracellular adhesion molecule (ICAM)– 1, E-selectin, IL-6, and IL-8 in human endothelial cells, as well as IL-8 in macrophages [107–110]. In line with these data, Lp(a) increases monocyte adhesion to human endothelial cells, and monocyte transmigration through the endothelial layer, early events in the development of atherosclerotic plaques [110,111]. In studies with E06 antibodies and apo(a)-mutated peptides that show reduced affinity for oxPL binding, all effects of Lp (a) on enhancing expression of adhesion molecules and cytokines were shown to be dependent on oxPL [109–111].

The role of Lp(a) in vascular inflammation has been studied in subjects with either high or low Lp(a) plasma levels. These subjects were not on statins, not smoking, and were matched for age, sex, and body mass index. They did not show differences in leukocyte levels or blood pressure [111]. Essentially, the only difference between these groups were the plasma Lp(a) levels. Using positron emission tomography/computed tomographic (PET/CT) imaging, subjects with high Lp(a) levels (average ~108 mg/dL) showed an increase in 18 F-fluorodeoxyglucose (FDG) uptake in the arterial wall of the carotid artery and ascending aorta in comparison to subjects with low Lp(a) (average ~7 mg/dL), reflecting an increase in arterial inflammation [111]. Normalized wall index of the carotid artery was not different between the groups as shown by magnetic resonance imaging (MRI) [111]. Subjects with high Lp(a) had high oxPL-apoB as well as oxPL-Lp(a) levels compared to the low Lp(a) group. Elegant ^99 m^Tc-labeling studies of autologous peripheral blood mononuclear cells (PBMCs) revealed an increase in PBMC accumulation in the arterial wall of the carotid and the aorta in subjects with high Lp(a) [111].

These studies for the first time provided solid evidence, in the human setting, that Lp(a) enhances arterial wall inflammation, presumably by enhancing monocyte entry. The latter could be the consequence of increased monocyte activation and/or endothelial activation. It has been shown in monocytes from patients included in the PET/CT study that an increase in plasma Lp(a) was accompanied by increases in C-C chemokine receptor 7 (CCR7), CD62L (L-selectin), the integrins CD11b, CD11c, and CD29 on the surface of monocytes, which reflect monocyte activation [111]. Monocytes from patients with high Lp(a) showed an increase in endothelial transmigration as well as cytokine secretion; the latter being mediated by oxPL on Lp(a) [111]. This likely explains the enhanced monocyte entry in the arterial wall in the subjects with high plasma Lp(a) levels.

Currently, antisense-based approaches to lower plasma apo(a) levels are in clinical trials. IONIS-APO(a)LRx decreases apo(a) plasma levels by ~72% and oxPL-Lp(a) levels at 85 days after injection compared to day 0 [45]. Interestingly, incubation of plasma from patients who received IONIS-APO(a)L_Rx_ with healthy aortic endothelial cells suppressed *ICAM-1, VCAM-1, monocyte chemoattractant protein-1 (MCP-1)*, and *IL-6* expression, compared to plasma from placebo treated patients, suggestive of suppression of endothelial activation by antisense oligonucleotides to apo(a) [110]. These data support the therapeutic potential of these drugs for decreasing CVD, in particular when CVD is driven by inflammation.

Further studies examining the monocyte activation phenotype by the same group have shown that elevated Lp(a) plasma levels in otherwise healthy individuals were associated with an increase in interferon α (IFNα) and IFNγ responsive genes in monocytes, compared to individuals with low Lp(a) [112]. These differences were exacerbated in monocytes from CVD patients with high Lp(a) plasma levels that also showed an increase in TNFα signaling pathways [112]. Additional studies showed that lowering Lp(a) levels by ~47% via an antisense-based approach (AKCEA-APO(a)-L_Rx_) in CVD patients led to suppression of IFNα, IFNγ, and Toll like receptor (TLR) responsive genes in monocytes [112]. AKCEA-APO(a)-L_Rx_ treatment also suppressed CCR2, CX3C chemokine receptor 1 (CX3CR1), and Toll like receptor 2 (TLR2) surface expression on monocytes, decreasing their trans-endothelial migratory capacity. These data support the findings in earlier studies [111] that Lp(a) enhances monocyte entry into atherosclerotic plaques, which may be mediated by direct effects on monocytes, and endothelial cells [110].

In the same study showing anti-inflammatory effects of AKCEA-APO(a)-L_Rx_ on monocytes [112], also monocytes from the ANITSCHKOW trial had been included. This trial included patients with high plasma levels of LDL-cholesterol and high Lp(a) (~80 mg/dL) [113]. While the PCSK9 antibody evolocumab lowered plasma LDL-cholesterol by ~60%, it reduced plasma Lp(a) by only ~14% [113]; and as a consequence did not affect arterial inflammation or inflammatory gene expression in monocytes [112,113]. These effects were attributed to Lp(a) reduction being only minimal after evolocumab treatment and reduction of LDL-cholesterol not affecting inflammation under conditions of high plasma Lp(a) levels [113]. A large percentage of patients with Familial Hypercholesterolemia (FH) also show Lp(a) levels > 50 mg/dL [114,115]. While PCSK9 antibodies reduce Lp(a) in these patients [114,115], the outcome of the ANITSCHKOW trial suggests that in the context of high Lp(a) levels (~80 mg/dL), reduction of Lp(a) by evolocumab is insufficient to suppress arterial wall inflammation or inflammatory gene expression in monocytes [112,113]. In a recent study, Santos et al. examined the long-term effects of evolocumab in patients with FH [116]. Evolocumab has similar efficacy in FH as in non-FH populations if patients have at least 1 normal *LDLR* allele. In this study, they did find lower than expected CV event rate of 2.7% per year vs. a range of 4–5% in previous studies [116].

However, subgroup results from the Studies of PCSK9 Inhibition and the Reduction of vascular Events (SPIRE) program, showed that statin-treated FH patients had a similar magnitude of risk reduction for hard cardiovascular events with the PCSK9 inhibitor bococizumab as did patients without FH, with no evidence of statistical heterogeneity [115]. The benefits of Lp(a) lowering using targeted apo(a) lowering treatments, in addition to LDL-cholesterol lowering therapies in this population are yet to be determined.

## 7. Concluding remarks

Collectively, it has been shown that Lp(a) enhances arterial inflammation, by stimulating monocyte entry, mediated by oxPL [110–112]. OxPL is a clear pro-atherogenic factor [93–95], and the small apo(a) isoform has high affinity for oxPL [93, 96, 97], and is associated with high Lp(a) levels that have been linked with CVD in Mendelian Randomization studies [36,100]. Additional work is needed to elucidate mechanisms linking Lp(a) to inflammatory phenotypes. The association of the small apo(a) isoform with high Lp(a) levels, and its high affinity for oxPL binding may lead to further understanding and discovery of pathways that link Lp(a) to inflammation. The role of the large apo(a) isoform in CVD remains understudied. Additional studies will be needed to elucidate whether the Lp(a) particle itself starts an inflammatory signal or existing inflammatory conditions drive the particle and its components to participate in pathways driving inflammation.

Since not all subjects with plasma Lp(a) levels > 50 mg/dL are at CVD risk, questions remain as to the additional risk factors that determine the pro-atherogenic and pro-thrombotic capacity of Lp(a). IL-1 induced inflammation may contribute to the effects of Lp(a) on CVD events [103]. Nonetheless, in this particular study [103], IL-1 may have increased plasma Lp(a) by increasing IL-6 expression, which complicates its interpretation.

Since several clinical trials on lowering plasma Lp(a) levels are ongoing [10, 45–49], questions remain as to which patients may benefit from Lp(a) lowering, especially since not all patients with elevated Lp(a) are at risk for CVD. These could include patients with elevated Lp(a) and high levels of inflammatory cytokines such as CRP, which is more routinely measured. In that regard, it would also be of interest to investigate whether anti-inflammatory drugs, such as canakinumab, or colchicine lower plasma Lp(a) levels, and as such are more effective in reducing CVD in patients with elevated Lp(a). One complication, as observed in a small trial with tocilizumab [73], is that several CVD patients are on statins, which have been associated with elevated Lp(a) [78]. Since tocilizumab treatment decreases plasma Lp(a) levels, and several lines of evidence indicate that Lp(a) is being produced downstream of IL-6 signaling, it will be of interest whether tocilizumab lowers plasma Lp(a) in the ASSAIL-MI trial [74] and whether this affects CVD events. Moreover, recent studies have shown that IL-6 inhibition with ziltivekimab in the Trial to Evaluate Reduction in Inflammation in Patients With Advanced Chronic Renal Disease Utilizing Antibody Mediated IL-6 Inhibition (RESCUE) has anti-inflammatory effects and decreases plasma Lp(a) dose-dependently. Ziltivekimab may be tested in a cardiovascular outcome trial [139]. Findings from the RESCUE trial suggest that its anti-inflammatory effects could be due to Lp(a) lowering.

## Figures and Tables

**Table 1:** Proteins Present on Immunoprecipitated Lipoprotein (a) Particles.

Detected Proteins (in addition to apo (a) and apoB100)	
ApoC1	Smallest apoliprotein in plasma. ApoC1 is associated with VLDL and HDL and acts on lipoproteins by inhibiting binding mediated by apoE to the LDL receptor, VLDL receptor, and LRP [117], and inhibiting lipoprotein lipase (LPL) [118], and cholesteryl ester transfer protein (CETP) [119]. It also binds to lipopolysaccharide, which increases inflammation, and protects against sepsis [120].
ApoA1	Is the main protein component of HDL. ApoA1 is involved in reverse cholesterol transport (RCT), i.e. the removal of cholesterol from macrophage foam cells in the arterial wall via ATP Binding Cassette Transporter A1 (ABCA1), transport in plasma, uptake by the liver and ultimate secretion into the bile. While mainly anti-inflammatory, pro-inflammatory effects of ApoA1 have also been reported [121].
ApoC2	Co-factor for LPL activity [122]
ApoC3	Inhibitor of LPL [123] and apoE mediated binding to LDL receptor and LRP [124]. Newly suggested role as pro-inflammatory apolipoprotein [125,126].
ApoE	Is associated with chylomicron remnants, VLDL, LDL, and HDL. Regulates VLDL and LDL clearance and contributes to HDL formation [127]. Binds lipopolysaccharide and protects against sepsis [128].
ApoF	Small apoliprotein associated with HDL, may have a role in lipid transfer between lipoproteins [129].
ApoA2	Secondary apolipoprotein contained in HDL particles, inhibits hepatic lipase to maintain HDL levels [130].
ApoD	ApoD is mainly associated with HDL and participates in lipid transport. It has a high affinity for arachidonic acid, and a diverse array of functions [131].
ApoJ/Clusterin	Associated with HDL [132]. Mainly role in Alzheimer’s Disease but crosses blood brain barrier [133].
ApoM	Is primarily expressed and secreted from the liver and present on HDL. ApoM is the main chaperone of sphingosine-1-phosphate (S1P) on HDL [134].
ApoC4	Mainly present on VLDL (80%) and also on HDL (20%) [135]. Linked to TG metabolism in mouse studies [136].
PAF-AH	Platelet activating factor – acetylhydrolase (PAF-AH) is present on LDL (70–83%) and HDL (11–30%) and hydrolyzes PAF-like oxidized phospholipids [137].
PON – 1	Paraoxonase-1 (PON-1) is mainly associated with apoA1 on HDL and inhibits LDL and HDL oxidation [138].
